# Wheel Replacing Pyramid: Better Paradigm Representing Totality of Evidence-Based Medicine

**DOI:** 10.5334/aogh.4341

**Published:** 2024-02-29

**Authors:** Colleen Aldous, Barry M. Dancis, Jerome Dancis, Philip R. Oldfield

**Affiliations:** 1 Nelson R. Mandela School of Clinical Medicine of the University of KwaZulu-Natal, Durban, South Africa; 2 Bioinformatics Consultant, Silver Spring, MD, USA; 3 Department of Mathematics, University of Maryland, College Park, MD, USA; 4 Scientific and Regulatory Consultant, Rigaud, Quebec, Canada

**Keywords:** evidence-based medicine, randomized control trial, ivermectin, COVID-19, quality of evidence, pyramid, wheel

## Abstract

**Background:**

Evidence-based medicine (EBM), as originally conceived, used all types of peer-reviewed evidence to guide medical practice and decision-making. During the SARS-CoV-2 Coronavirus disease (COVID-19) pandemic, the standard usage of EBM, modeled by the Evidence-Based Medicine Pyramid, undermined EBM by incorrectly using pyramid levels to assign relative quality. The resulting pyramid-based thinking is biased against reports both in levels beneath randomized control trials (RCTs) and those omitted from the pyramid entirely. Thus, much of the evidence was ignored. Our desire for a more encompassing and effective medical decision-making process to apply to repurposed drugs led us to develop an alternative to the EBM Pyramid for EBM. Herein, we propose the totality of evidence (T-EBM) wheel.

**Objectives:**

To create an easily understood graphic that models EBM by incorporating all peer-reviewed evidence that applies to both new and repurposed medicines, and to demonstrate its potential utility using ivermectin as a case study.

**Methods:**

The graphics were produced using Microsoft Office Visio Professional 2003 except for part of the T-EBM wheel sunburst chart, which was produced using Microsoft 365 Excel. For the case study, PubMed® was used by searching for peer-reviewed reports containing “ivermectin” and either “covid” or “sars” in the title. Reports were filtered for those using ivermectin-based protocols in the treatment of COVID-19. The resulting 265 reports were evaluated for their study design types and treatment outcomes. The three-ringed graphical T-EBM wheel was composed of two inner rings showing all types of reports and an outer ring showing outcomes for each type.

**Findings-Conclusions:**

The T-EBM wheel avoids the biases of the EBM Pyramid and includes all types of reports in the pyramid along with reports such as population and mechanistic studies. In both early and late stages of medical emergencies, pyramid-based thinking may overlook indications of efficacy in regions of the T-EBM wheel beyond RCTs. This is especially true when searching for ways to prevent and treat a novel disease with repurposed therapeutics before RCTs, safety assessments, and mechanisms of action of novel therapeutics are established. As such, T-EBM Wheels should replace the EBM Pyramids in medical decision-making and education. T-EBM Wheels can be expanded upon by implementing multiple outer rings, one for each different kind of outcome (efficacy, safety, etc.). A T-EBM Wheel can be created for any proprietary or generic medicine. The ivermectin (IVM) T-EBM Wheel displays the efficacy of IVM-based treatments of COVID-19 in a color-coded graphic, visualizing each type of evidence and the proportions of each of their outcomes (positive, inconclusive, negative).

## Introduction

**Figure 1:**
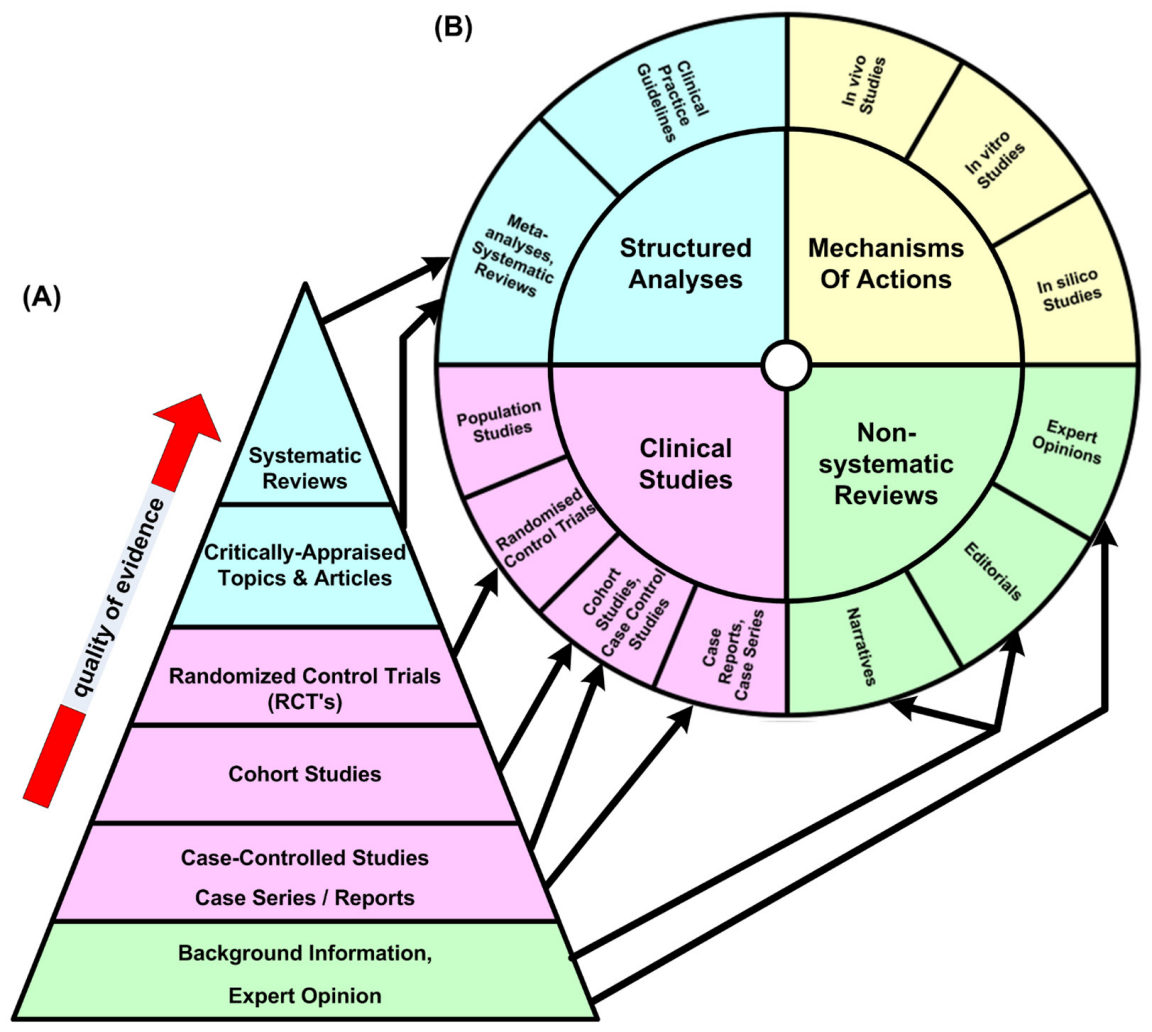
Correspondence of traditional QoE Pyramid with T-EBM Qualitative Wheel. **(A)** QoE Pyramid. The pyramid represents a purported hierarchy of ever-increasing quality. The color of each level of the pyramid was chosen to match its corresponding section of the wheel. **(B)** T-EBM Qualitative Wheel. Rings show various types of reports. Arrows link each level of the pyramid with its corresponding section in the outer ring of the T-EBM Qualitative Wheel.

We introduce the Totality of Evidence-Based Medicine (T-EBM) Wheel, a novel paradigm designed to support and improve medical education and the decision-making process of evidence-based medicine (EBM). By “Totality,” we refer to both the totality of the study design types of peer-reviewed reports and the totality of such reports for each given type. The T-EBM wheel is proposed as a replacement for the traditional EBM Pyramid (Figure 1), which, through the addition of the quality arrow from the bottom to the top of the pyramid, has become what we will refer to as a Quality of Evidence (QoE) Pyramid.

The QoE Pyramid prioritizes randomized control trials (RCTs), which are expensive and time-consuming when demonstrating the safety and efficacy of medications. During the urgency of a pandemic, the limitations of conducting RCTs for novel treatments are exacerbated in such a rapidly evolving situation. The QoE Pyramid is also not consistent with the original intent of EBM.

EBM is a process that initially emphasized the necessity of basing medical decision-making on all types of peer-reviewed scientific research to eliminate reliance on intuition and uncritical adherence to treatment trends. EBM gained its current prominence in medical research and education during the 1990s as a result of the influential publications by David Sacket and his colleague Gordon Guyatt [1,2,3]. The ubiquitous QoE Pyramid arose out of their [3] hierarchical grading system and was solely based on study design. Placing a study in a pyramidal hierarchy, however, only assigns the relative certainty of evidence, namely the power to make inferences, not the relative quality of that study. The school of thought that pyramid levels relate to quality (pyramid-based thinking) and not certainty, however, facilitated the interpretation and application of EBM to change dramatically. Only those reports at the level of RCTs and above have been deemed worthy of clinical decision-making and policy recommendations. This has resulted in poorly designed or poorly executed RCTs being accepted as high quality, not because of their actual scientific merit, but because of their position in the QoE Pyramid. Importantly, although the results of a single observational study may have less certainty than those of a single RCT, *a priori*, quality studies of the same disease should yield similar results independent of study design. And in fact, they do: “On average, there is little evidence for significant effect estimate differences between observational studies and RCTs, regardless of specific observational study design, heterogeneity, or inclusion of studies of pharmacological interventions” [4]. A review describing studies from the 1990’s also reported that “observational studies got similar results to RCTs” [5]. Thus, ignoring real-world evidence (RWE) and other reports not found in the QoE Pyramid undermines the original intent of EBM to use signals from all types of study designs.

### T-EBM Wheel is a Proper Superset of QoE Pyramid

Figure 1 compares the composition of the T-EBM Qualitative Wheel with that of a QoE Pyramid. The arrows show the correspondence between all types of reports in the pyramid with a subset of those in the wheel. It is clear from the figure that the T-EBM Wheel offers a more holistic and inclusive framework that is fully consistent with EBM. It integrates a wider range of peer-reviewed evidence, adding observational studies, mechanistic studies, and expert opinions, to ensure a comprehensive representation of medical research. This approach addresses the limitations of the QoE Pyramid by providing a more balanced and complete overview of available evidence, and it addresses the false quality bias of the hierarchical QoE Pyramid by providing a non-hierarchical categorical structure where no sector is privileged over another.

During the early stages of the CoV-2 Coronavirus disease (COVID-19) pandemic, rapid decision-making was constrained by pyramid-based thinking, which derives from the hierarchical structure of the QoE Pyramid and limits consideration to RCTs. Consequently, there were many ignored studies for repurposed drugs using a variety of other study designs, but no methods were available to obtain guidance for assessing their overall results. For example, much of that evidence indicated ivermectin (IVM)-based therapies as effective agents against COVID-19, yet governmental and medical literature justified downplaying its role is consistent with pyramid-based thinking. If the hierarchical structure of the QoE Pyramid was not helpful in that situation, might there be a different structure that would be? It was this deficiency that prompted the development of a non-hierarchical categorical all-inclusive structure of the T-EBM Wheel that avoids the quality bias of the QoE Pyramid as well as its limited variety of study designs. Thus, the wheel includes all the RWE as well as RCTs, with every type of study design considered equally. With an outer ring that displays the proportions of outcomes when applied to a specific medicinal product, as demonstrated in our case study, the T-EBM Wheel can serve as a guide for rapid and informed medical decision-making and education during a quickly evolving pandemic situation, especially when RWE for repurposed drugs is often much more available than RCTs.

As proof of concept, we examined applying the T-EBM Qualitative Wheel to the case of treating COVID-19 with IVM-based regimens, many of which included multidrug and supplementary components. We achieved this by adding a third and outermost ring to display the relative proportions of efficacy outcomes of IVM-based treatments. This additional ring demonstrates the many types of reports aside from RCTs that contain information about IVM-based therapies that could have been used to guide medical decision-making. This further underscores the utility of T-EBM Wheels by academics, researchers, and government agencies, especially for repurposed drugs during emergencies such as pandemics.

A newspaper version of this article appeared in Trial Site News [6], which was subsequently reported in BizNews [7]. The analysis presented in this paper extends and refines the work reported in those articles by the same authors.

## The Case Study: Construction of an IVM T-EBM Wheel

Selecting IVM as a case study for EBM during the pandemic was a prudent choice due to its controversial status in the treatment of COVID-19. This drug, originally used for parasitic infections, garnered widespread attention and divergent views regarding its efficacy against the virus. Using IVM as a focal point provides a valuable opportunity to scrutinize and apply rigorous scientific methods and ethical standards in evaluating its effectiveness. This scenario highlights the importance of relying on robust, peer-reviewed research to guide clinical decisions, particularly in a rapidly evolving public health crisis. It also underscores the critical role of EBM in distinguishing between well-supported treatments and those promoted without sufficient scientific backing, thus contributing to more informed and effective healthcare practices.

### Materials And Methods

#### Unbiased Search for Reports of IVM-based COVID-19 Treatments

The PubMed® database is internationally recognized as the most comprehensive source of published medical research literature. For proof of concept, an actuary with no conflicts of interest was hired for this particular study to conduct a thorough PubMed® search for reports prior to October 2022 containing “ivermectin” and either “covid” or “sars” in the title. Such a search would retrieve both literature evaluating the efficacy of ivermectin-based therapies against COVID-19, as well as those that did not. There would be no selection bias on outcomes based on this simple search. Any further qualifying criteria may have created a bias. We suggest that simpler Boolean search strings are preferable to avoid unintentional selection biases. By restricting searches to objective keywords and phrases, such as the name of the disease and its treatments, and by omitting consideration of treatment outcomes or patient demographics, an unbiased selection process can be fostered. To further avoid bias, the obtained reports were not directly compared or individually evaluated for quality other than that they were peer-reviewed. Using only PubMed® for the searches, we did not capture relevant literature found only in other sources. Nevertheless, our search did find substantial evidence on the use of IVM-based therapies from studies with disparate designs. All reports used in this study are available as free full texts either from PubMed® or online.

#### Data source and extraction method

The PubMed® catalogue [8] was accessed online on the first working day of the months from June to October 2022, covering reports listed in the catalog up to the end of September 2022. To improve the credibility of the results, only published articles were selected, as they ostensibly would have been subjected to rigorous and objective peer-review processes. Filtering those reports for free full texts selected whole articles freely available from PubMed®. The researchers chose to use publicly available data so that the study findings could be independently replicated and verified. A list of the search parameters is shown in Table 1.

**Table 1:** Parameter values used in primary search of PubMed® Catalog.


PARAMETER	VALUE

Date – Published	Up to 2022 September 30

Text Word	(ivermectin) and ((covid) or (sars))

Filter: “TEXT AVAILABILTY” boxes checked	“Abstract”, “Free full text”, “Full text”


Each report was counted as one data point with the following fields: PMID (unique identifier), title, authors, first author, journal, and publication date. The PubMed® results were imported into the citation manager, Mendeley. This allowed each article to be loaded, and the abstract reviewed to understand the nature of the study, its findings, and conclusions.

To verify if we missed any clinical studies by using the filter “Free full text,” a supplementary search was done to identify relevant clinical studies listed on PubMed®, even if the full articles were only accessible elsewhere. The PubMed® “Date – Published” and “Text Word” parameters remained the same. The supplemental filters are listed below in Table 2.

**Table 2:** Parameter values used in supplementary search of PubMed® Catalog Parameters for supplementary search of PubMed® Catalog to find clinical articles whose “Full Text” would only be available from non-PubMed® sources.


PARAMETER	VALUE

Date – Published	Up to 2022 September 30

Text Word	(ivermectin) and ((covid) or (sars))

Filter: “TEXT AVAILABILTY” boxes checked	“Abstract”, “Full text”

Filter: “ARTICLE TYPE” box checked	“Clinical Trial”


The supplementary search yielded only two additional citations not found in the first extract. The titles of those two articles were used in internet searches, and their free reports were downloaded from sites other than PubMed®. The citations of the combined primary and supplementary searches were saved to a *.csv* file for further analysis.

**Figure 2:**
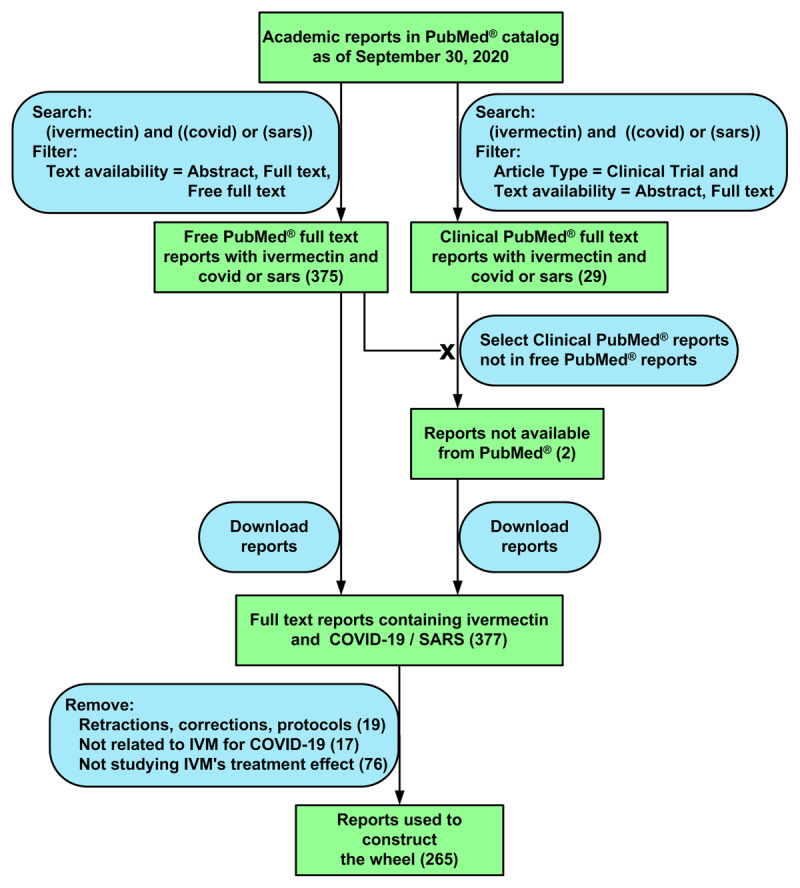
Flowchart for extracting citations about published IVM-based COVID-19 treatments from PubMed®. The reports of the citations were used to construct an IVM T-EBM Wheel. The left column shows the initial extraction which obtained 263 citations. The column on the right shows the extraction of the two clinical full text citations whose text were only available from sources other than PubMed®. The number of citations at a particular step are shown in parentheses. The “X” mark indicates removal of clinical citations from the right column previously found in the left column.

The data was processed by removing PubMed® entries not relevant to this research (Figure 2). The figure was produced using Microsoft Office Visio Professional 2003.

#### Data classification

All 265 reports after filtering were classified into four types (each with its own subtypes):

1. Structured analyses (systematic reviews, meta-analyses, and clinical guidelines) use structured approach to analyze, integrate, and critically appraise primary evidence.
2. Clinical studies (RCTs, cohort studies, case-control studies, case series, case reports, population studies) are primary studies with human subjects; population studies are post hoc analyses of data for a drug provided to a population, but not as part of a clinical experiment.
3. Mechanisms-of-action (*in-silico*: computer-based studies, *in-vitro*: lab-based studies and *in-vivo*: animal studies) are all studies without human subjects.
4. Non-systematic reviews (narrative reviews, editorials, and expert opinions).

Outcomes were determined by statements about efficacy of treatments in the reports. For all but the non-systematic reviews, such statements were obtained from the conclusion sections, even in cases where the conclusions were contradicted by, or not supported by, the findings. For the non-systematic reviews, the outcomes were inferred from the abstract, if present, or the body of the text when not. Outcomes were rated as follows:

1. Positive: the IVM-based regimen was effective for treating COVID-19 (or, for mechanisms-of-action, there was a plausible reason for the effectiveness of IVM-based treatments for COVID-19).
2. Inconclusive: the evidence was insufficient or conflicting or the sample sizes were too small to show significant differences though the results may be promising; more research is required.
3. Negative: the IVM-based regimen was ineffective for treating COVID-19 (or, for mechanisms-of-action, there was a plausible reason for the ineffectiveness of IVM-based treatments for COVID-19).

The outcomes were visually displayed by adding a color-coded outermost ring to the T-EBM Qualitative Wheel to create the three ring IVM T-EBM Wheel.

#### Construction of Figures

**Figure 3:**
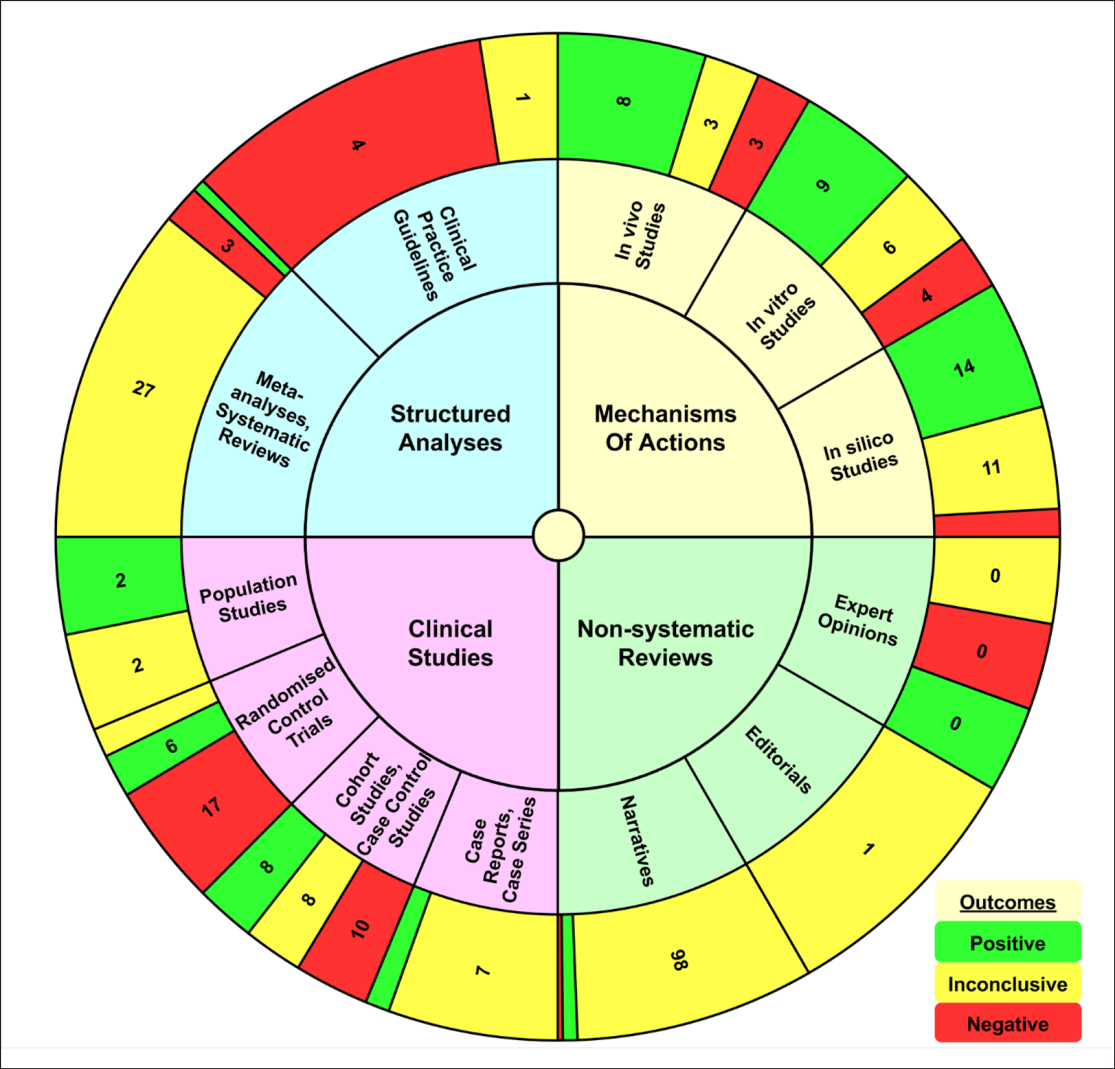
IVM T-EBM Wheel for the efficacy of IVM-based treatments of COVID-19. The inner ring of the wheel catalogues reports into four types: 1) mechanisms of action and 2) clinical studies, both of which are sources of primary evidence, and 3) structured analyses of primary data and 4) reviews, both of which are the secondary reports based on the primary evidence. Each inner ring section is disaggregated into several middle ring sections with the same coloring. In turn, each middle ring section is disaggregated into three outer ring sections by the outcomes of its reports, namely: “positive,” “inconclusive,” “negative.” The legend in the lower right-hand corner of the figure shows the color associated with each of the outcomes. For each middle ring section, the size of its three outcome sections in the outer ring is proportional to its number of reports in the literature and the order of those three outcome sections is clockwise from largest to smallest and not by outcome. Data from peer-reviewed published reports listed in PubMed® and searched for “ivermectin” and for either “covid” or “sars”. The number displayed in each outer ring section is the number of its reports. Numbers are omitted when the section is too narrow for display (see Table 3 for all values).

The QoE Pyramid (Figure 1a) and the Flow Chart (Figure 2) were produced using Microsoft Office Visio Professional 2003 (Visio). The T-EBM Qualitative Wheel (Figure 1b) and the IVM T-EBM Wheel (Figure 3) were produced as Visio modifications of Microsoft 365 Excel (Excel) Sunbursts. An application using Excel Visual Basic for Applications was coded to automate the sunburst creation and initial formatting of the T-EBM Wheels.

**Table 3:** Outcomes of treating COVID-19 with IVM-based protocols.


REPORT TYPE	SOURCE OF EVIDENCE	TOTAL	POSITIVE	NEGATIVE	INCONCLUSIVE

**Structured analyses**	Clinical practice guidelines	5	0	4	1

	Meta-analyses, Systematic reviews	31	1	3	27

**Clinical studies**	Randomized control trials (RCTs)	27	6	17	4

	Cohort studies, Case control studies	22	6	9	7

	Case reports, Case series	8	1	0	7

	Population studies	4	2	0	2

**Non-systematic Reviews**	Narratives	106	6	2	98

	Expert opinions	1	0	0	1

	Editorials	0	0	0	0

**Mechanisms of action**	*In-silico* studies	28	14	3	11

	*In-vitro* studies	19	9	4	6

	*In-vivo* studies	14	8	3	3

**Total**		265	53	45	167


### Results

Searching the PubMed® database yielded 377 articles. Excluded from the study were 19 retractions, corrections, and protocols, 17 articles not related to IVM or COVID-19 and 76 articles not analyzing the efficacy of IVM-based treatments of COVID-19. Therefore, 265 out of 377 articles found, i.e., 70%, were included in the study.

#### Proof of Concept of a T-EBM Wheel for IVM Efficacy in Treating COVID-19

A T-EBM Wheel for the efficacy of IVM-based COVID-19 treatments was created as a proof of concept. The graphic aims to provide medical educators and decision makers with an easily understood visual aid. The outer ring of the IVM T-EBM Wheel (Figure 3) displays the summary of the outcomes (Table 1). The width of a section in the outer ring also presents a visual representation of the relative numbers of outcomes for reports in that section. Color-coding the types of outcomes in the outer ring facilitates visualization of indicators of areas of clinical uncertainty.

Around a fifth of the research reports had positive outcomes, and another fifth had negative ones. Most of the reports had inconclusive results, sometimes because no major or systematic benefits from the treatment were observed and other times because sample sizes were too small to provide statistical significance, mostly for positive findings. About two-thirds of the reports are reviews and mechanisms of action. About one paper in eight is a structured analysis (systematic reviews, meta-analyses, and clinical guidelines). The remaining fifth of the reports are the 61 clinical studies (RCTs, cohort studies/case-controlled studies, case reports/case series, and population studies). Of the 27 RCT reports, the protocols with more successful outcomes used higher dosages, more elaborate combinations of other drugs and adjuncts, and longer regimens than those with less successful outcomes. Additionally, the aforementioned successful protocols often included taking IVM with fatty meals. Regardless of outcomes, every one of the reports confirmed the absence of serious adverse events consistent with previous studies using IVM for the treatment of other human diseases [9,10,11,12,13].

## Discussion

We have constructed an easily understood graphic that visualizes report outcomes and is consistent with EBM by including all types of peer-reviewed evidence without favoring or implying the quality of any particular type of evidence. While our IVM T-EBM Wheel does include numbers for outcomes, those numbers are not to be used for comparison. Under the most favorable circumstances, they can only be used as indicators of outcomes, especially because individual reports are not evaluated for quality and the conclusions in some reports are not supported by their findings. In addition, a T-EBM Wheel, by its very nature, will result in some reports appearing in multiple sections, namely in primary and secondary reports, as well as non-systematic reviews. Each T-EBM Wheel presents a totality of evidence to encourage integration of results from multiple sections. As an example, mechanistic studies should help explain and predict the results of clinical trials. The aim of T-EBM Wheels is not to weigh the relative merits of individual sections or reports but to display all the sections in an objective manner. However, it is crucial to understand that the objectivity of the selection process does not reduce the subjectivity of the individual reports themselves.

As shown in Figure 1, each T-EBM Wheel contains a proper superset of the data and information found in the QoE Pyramid while omitting its misleading quality hierarchy. Pyramid-based thinking ignores the evidence in the bottom levels of the pyramid, and the population and mechanisms of action studies found in T-EBM Wheels. The overemphasis on RCTs and their meta-studies when evaluating all therapeutics, including repurposed ones with established safety profiles, has contributed to much valuable evidence and important signals of clinical benefit being overlooked or neglected by medical decision-makers. Their resulting recommendations have often delayed and/or prevented effective treatments using repurposed drugs. Thus, the T-EBM Wheel replacing the QoE Pyramid as an application tool for medical professionals and decision-makers will comparatively increase scope and decrease bias. During an emergency, ignoring RWE can result in failing to repurpose known drugs. Unfortunately, medical training and media discourse have ingrained in both medical professionals and the public that, *a priori*, RWE is of low quality and RCTs are of high quality. This has been embodied in a medical textbook as: “If the study was not randomized, we would suggest that you stop reading it and go on to the next article” [14]. Ignoring RWE also contravenes the 21^st^ Century Cures Act where the US government states that for the purpose of the Act, RWE “means data regarding the usage, or the potential benefits or risks, of a drug derived from sources other than randomized clinical trials,” and mandated that RWE should be used “to help support the approval of a new indication for a drug [previously] approved,” that is, for repurposing drugs [15].

In 2021, Deaton, the winner of the 2015 Nobel Prize in Economics, and Cartwright analyzed RCTs [16] and determined they had serious limitations including failure to balance confounders and finding little practical value of unbiasedness compared to precision. They highlight their conclusion that “RCT results can serve science but are weak ground for inferring ‘what works’ [clinically].”

By the mid-1990’s, the social sciences had already come to a similar conclusion about overvaluing RCTs. Walach et al [17]. promoted the use of

“a multiplicity of methods, [with] different designs, counterbalancing their individual strengths and weaknesses to arrive at pragmatic but equally rigorous evidence which would provide significant assistance in clinical and health systems innovation. Such evidence would better inform national health care technology assessment agencies and promote evidence-based health reform.” In the main body of their paper, Walach et al. declare: “Rather than postulating a single ‘best method’ this view acknowledges that there are optimal methods for answering specific questions, and that a composite of all methods constitutes best scientific evidence. […] The important point is not whether a study is randomized, but whether it uses a method well suited to answer a question and implements this method with optimal scientific rigor. […] Methods that are high in internal validity, such as placebo controlled RCTs […] tend to be lower in external validity. […] Thus, their results need to be balanced by large and long-term observational studies which document the use, safety, and effectiveness of the intervention in clinical practice” [17].

Walach et al. also developed the Circular Model for types of studies to replace a hierarchy of evidence that overvalued RCTs [17]. It is similar in shape and spirit to our T-EBM Wheel. Our wheel, however, displays additional types of evidence and adds proportional representation of the outcomes of that evidence. Both the Walach et al. Circular Model and our T-EBM Wheel will lead clinicians and decision-makers to broader bodies of useful information than pyramid-based thinking. Only our wheel, however, includes the outcomes of the totality of evidence to guide frontline medical professionals who are making bedside decisions that should integrate RWE and RCTs when available with their personal observations as they formulate individual treatments and collectively advance a “community standard of care.”

Use of the QoE Pyramid also raises the ethical question: Is it fair to the patient to only look at the evidence from a restricted set of methods? In medical ethics, clinical equipoise occurs “if there is genuine uncertainty within the expert medical community […] about the preferred treatment” [18]. A T-EBM Wheel can display possible clinical equipoise; when it does, there should be different responses depending upon the type of responder. Medical practitioners should be guided by the positive signals for treatment and by the negative signals for what to avoid. Clinical researchers should perform further studies to improve efficacy and/or help select among regimens. Governmental and medical authorities should initially present the collection of different findings while remaining neutral concerning any pronouncements on treatments and then modify their neutrality accordingly as new reports decrease the degree of clinical equipoise. The bases for those pronouncements should be the totality of evidence — clinical studies (both observational studies and RCTs), structured analyses, mechanistic studies, and non-systematic reviews.

The collective outcomes of anecdotal evidence along with other RWE, have strong plausibility. They should not be dismissed as lacking credibility as commonly done today [19]. In the case of repurposed drugs with known safety profiles, T-EBM Wheels in real time with strong positive outcomes will indicate what RCTs are required, or even ethical, to quantify efficacy. Many of the reports in our IVM T-EBM Wheel were retrospective ones where IVM was included in therapeutic regimens consistent with the ethical obligation of the frontline medical professionals to treat patients while doing no harm. In an emergency with a positive mortality rate and a T-EBM Wheel that indicates possible clinical equipoise, one could argue that medical researchers conducting an RCT with a placebo control arm would be unethical. In the case of the COVID-19 pandemic, the only initial studies truly required would have been dosing to effect with repurposed drugs and their adjuncts, as suggested by the reports with positive outcomes/signals in our IVM T-EBM Wheel.

### Scenario For Using T-EBM Wheels of Known Drugs in Emergencies

In an emergency’s early stages, “time and cost to develop a new indication for an existing drug may be significantly less compared to developing a new drug from scratch because: Most of the non-clinical drug development has already been done including chemistry, manufacturing and control, animal toxicology and clinical pharmacology. [And] there is clinical data on safety in a population that may be relevant to the novel use” as noted in an FDA report [20]. Thus, medical professionals, authorities, and decision-makers should prioritize the repurposing of known medications. Retrospective reviews of existing data for similar diseases would be an appropriate first step in estimating therapeutic potential. Early signals of efficacy might also be derived from previous *in-vitro, in-vivo*, and *in-silico* studies. Such reviews and mechanistic studies may, at worst, lead to exploring drug protocols that later prove to be ineffective (i.e., false positives/type 1 errors). Such errors, however, would not pose additional safety risks. Dosing to effect for such drugs is particularly useful to frontline medical professionals because they already have the regulatory approvals lacked by novel drugs and it tremendously enhances the likelihood of successful treatment. Such professionals should be relied upon for their clinical experience as they directly interact with patients and are responsible for making treatment decisions. Patients, once assured a treatment is safe and potentially effective, can give informed consent to be so treated. The primary objective is to cause no harm while anticipating positive outcomes and documenting any adverse ones. By closely monitoring patients, treatments can be modified accordingly and widely disseminated through the publication of case studies and case series, which in turn can lead to higher certainty, and more importantly, more effective treatments.

As case studies and other observational studies become available, they provide early signals of efficacy and should not be ignored. Indeed, they should be used to design clinical protocols for new observational studies and RCTs. Signals from different trials for different drug dosages and adjuncts could be used to design protocols that combine them in novel ways and evaluated in clinical studies. It is important to note that the variety of protocols using well-known therapeutics in observational studies is far larger than that for RCTs. This variety gives frontline medical professionals the flexibility to assess and customize treatment regimens as they search for increasingly positive results.

Governmental and medical authorities monitoring the results of the frontline medical professionals should recommend experimental usage as appropriate on a broader scale while knowing that drug regimens might not be effective but at least should be safe. At an institutional level, separate T-EBM Wheels for efficacy, or a single wheel with a separate ring for each drug, should be created early in a pandemic, using all data available. Such wheels would organize the data visually and show the existence of efficacy reports with positive, inconclusive, and negative outcomes. For all outcomes, it is important to remember that the effectiveness of drugs is often highly dependent upon the specifics of a regimen and the demographics of the patients. Analyzing those reports may yield additional information useful for therapeutic decision-making, including dosage regimens, safety considerations, mechanisms of action and demographics.

Regularly updating a T-EBM Wheel will provide a timely visual summary of the totality of evidence developing around the efficacy of treatment protocols. Looking at a T-EBM Wheel, researchers and decision-makers should be alert for positive outcomes and signals being visible throughout its entirety and guide their research and pronouncements accordingly. Concurrently, a sponsor could initiate the appropriate regulated studies to add a new indication to the label of a repurposed drug. This process would avoid the selective bias resulting from pyramid-based thinking and the resulting lag in finding solutions. “The goal must [always] be actionable data [in a timely fashion] — data that are sufficient for clinical and public health action that have been derived openly and objectively and that enable us to say, Here’s what we recommend and why” [21].

Such goals are often difficult to achieve with RCTs alone since, “RCTs often take years to plan, implement, and analyze reduce[s] the ability of RCTs to keep pace with clinical innovations; new products and standards of care are often developed before earlier [RCT] models complete evaluation. These limitations also affect the use of RCTs for urgent health issues, such as infectious disease outbreaks, for which public health decisions must be made quickly on the basis of limited and often imperfect available data” [21]. Frontline medical professionals regularly prescribe drugs outside the scope of their initially approved usage without requiring new approvals; a practice referred to as “off-label use.” Before making therapeutic decisions, healthcare professionals should refer to the existing literature to determine the safety profile and potential efficacy of a drug for a specific disease.

It is important to note that we have provided a framework, but not a prescriptive guide, where medical decision-makers could effectively use T-EBM Wheels as an aid for more rapidly repurposing drugs during pandemics.

### The Use of T-EBM Wheels During the COVID-19 Pandemic

T-EBM wheels for several repurposed drugs would be useful to guide medical decision makers during any pandemic. We have looked at the role of an IVM T-EBM wheel for treating COVID-19. Prior to that pandemic, IVM already had an excellent human safety record, both during its development (including RCTs and observational studies) and extensive usage (over 4 billion doses since the 1980’s) [9,10,11]. That safety record was available from the World Health Organization (WHO) VigiAccess database [12]. Both the initial and subsequently tried IVM-based regimens for the treatment of COVID-19 were well within the safety range covered in a 2002 Phase 1 study [13]. In fact, the press release [22] for the 2015 Nobel Prize in Physiology or Medicine cited “the importance of ivermectin for improving the health and wellbeing of millions of individuals” with “limited side effects.”

By 2020, there already were *in vitro* studies demonstrating IVM had potent antiviral activity against COVID-19 and other RNA viruses [23]. Additional reports have demonstrated that IVM has multiple mechanisms of action for different indications including its anti-inflammatory properties [24], which reduce or eliminate the cytokine storms that cause so many COVID-19 patients to succumb prematurely [25,26,27]. By December 2020, a retrospective study of Florida hospitalized patients with severe COVID-19 found a 40% reduction in mortality rates for those treated with IVM plus the standard of care (SOC) compared to those treated only with SOC [28]. More importantly, IVM was shown to greatly improve SpO_2_ (a measure of blood oxygen saturation) of patients with severe COVID-19 within 24 hours after treatment so that they didn’t need subsequent hospitalization [29,30,31] in contrast to the well-established SOC of hospitalization with no improvement of SpO_2_ for that same time period.

All these studies, however, had only localized effects on treatment and prevention. Pyramid-based thinking created a bias against the RWE and mechanistic studies, downplaying and/or ignoring those positive study results. In the NIH guidelines of April 2022 [32], the universal recommendation against the use of IVM for COVID-19 was primarily informed by four RCTs, one of which [33] was significantly underdosed and the other three [34,35,36] had significant protocol violations [37]. None of those studies, each with its own particular protocol and demographics, used adjuncts or had seriously ill patients. Thus, the NIH recommendation, which extrapolated beyond the data of those flawed studies, seems to have been unwarranted.

It is evident from our IVM Wheel that at least by 2022 there were positive indications of efficacy across all types of reports. Statistically positive results could have provided suggestions for using IVM-based regimens, and both the positive and inconclusive results with positive signals, for designing new IVM-based protocols. Negative results would have also suggested aspects of treatment regimens to modify or avoid. Even the aforementioned flawed studies could have suggested aspects of protocols to avoid, while their positive findings, when contradicting their negative conclusions, could have suggested aspects of protocols producing positive effects. The use by decision-makers early on of an IVM T-EMB Wheel (e.g., Figure 3) might have been a useful guide for suggesting treatments during the COVID-19 pandemic.

### The T-EBM Wheel as a Comprehensive Model

To interpret the T-EBM Wheel, readers should view it as a comprehensive model that integrates various types of medical evidence. The wheel is divided into segments, each representing a different kind of evidence, such as randomized controlled trials, observational studies, case reports, and expert opinions. These segments are interconnected, illustrating how all forms of evidence contribute to a holistic understanding of medical research.

Each segment of the wheel is labeled for easy identification. The central hub of the wheel underscores the core principle of inclusive evidence evaluation, reminding readers that all types of evidence have value in the context of evidence-based medicine.

This model allows for a more nuanced and inclusive approach to evaluating medical evidence, acknowledging the strengths and limitations of each type of study. By considering the full spectrum of evidence, healthcare professionals and researchers can make more informed decisions that reflect the complexity of medical science.

### Limitations

The major limitation of this study is that while some guidelines are provided for using a wheel, a prescriptive guide is not provided. Users must decide for themselves what information they want to use in their frontline medical activities, and what information they want to follow up on in their policy or research decision-making. Limitations in constructing our IVM T-EBM Wheel include that reports were not screened by quality or for conflicts of interest, for content of protocols other than it included IVM or for the strain(s) of virus being addressed. Reports were limited to those found in PubMed® and we arbitrarily decided to choose the outcomes in the conclusions even when contradicted by the findings. Wheels do not include information about sample sizes, magnitude of statistical significance or other attributes, as the model itself is not intended to compare or evaluate individual reports. Wheels do not indicate how report results might be combined or generalized.

With a new drug where there is only limited literature available, a T-EBM wheel would not yield much information. It is particularly advantageous for repurposing, where there already is a body of literature which includes known safety profile, roll-out potential and possible indications of efficacy. However, developments of new drugs are often not published, making it more difficult to evaluate efficacy, safety and mechanisms of action using a wheel.

## Conclusions

When in the course of the COVID-19 pandemic, our desire for a more effective medical decision-making process for repurposed drugs led us to develop an alternative to the QoE Pyramid for EBM. The resulting T-EBM Wheel using IVM as an example is an easily understood color-coded graphic that provides non-hierarchical unbiased information about the types, outcomes, and numbers per outcome of peer-reviewed research reports. Most notably, it displays disparate research results as a unified body of work. It draws the user’s attention to RWE, mechanisms of action and non-systematic reviews, in addition to the RCTs and structured analyses used almost exclusively now. The T-EBM Wheel is highly adaptable; by adding extra outer rings, it can also display safety outcomes, disaggregated demographics, multiple drugs, and disaggregated severity. During emergencies and before development of agreed upon successful treatments, frontline medical professionals need wide latitude to experiment with repurposed drugs and treatments known to be safe. This medical analogue of brainstorming generates many hypotheses for selecting aspects of successful protocols for further treatment and research. T-EBM Wheels facilitate such activity by providing wide varieties of useful information. Ideally, frontline medical professionals can integrate such information with their personal observations as they formulate individual treatments. They, in conjunction with researchers, can then collectively advance a ‘community standard of care’ not driven solely by a limited evidence review, government agencies or medical authorities.

In contradistinction, the hierarchical QoE Pyramid omits the population studies, mechanisms of action, editorials and narratives found in T-EBM Wheels resulting in pyramid-based thinking that significantly limits the scope of information considered for further analysis and thereby impedes medical progress. This is especially harmful during emergencies and undermines EBM. Clearly, there is a need for a more robust, inclusive, adaptable, and unbiased approach in medical schools, clinical practice, and evidenced-based medicine in general; an approach exemplified by our T-EBM Wheel.
